# BUSIS: A Benchmark for Breast Ultrasound Image Segmentation

**DOI:** 10.3390/healthcare10040729

**Published:** 2022-04-14

**Authors:** Yingtao Zhang, Min Xian, Heng-Da Cheng, Bryar Shareef, Jianrui Ding, Fei Xu, Kuan Huang, Boyu Zhang, Chunping Ning, Ying Wang

**Affiliations:** 1 School of Computer Science and Technology, Harbin Institute of Technology, Harbin 150001, China; yingtao@hit.edu.cn; 2 Department of Computer Science, University of Idaho, Idaho Falls, ID 83402, USA; shar0416@vandals.uidaho.edu; 3 Department of Computer Science, Utah State University, Logan, UT 84322, USA; fei.xu@aggiemail.usu.edu (F.X.); kuan.huang@aggiemail.usu.edu (K.H.); 4 School of Computer Science and Technology, Harbin Institute of Technology, Weihai 264209, China; jrding@hit.edu.cn; 5 Institute for Modeling Collaboration and Innovation, University of Idaho, Moscow, ID 83844, USA; boyuz@uidaho.edu; 6 Department of Ultrasound, Medical College of Qingdao University, Qingdao 266003, China; ningchunping1222@163.com; 7 Department of General Surgery, Hebei Medical University, Shijiazhuang 050017, China; dina513@163.com

**Keywords:** breast ultrasound (BUS) images, segmentation, computer-aided diagnosis (CAD), benchmark

## Abstract

Breast ultrasound (BUS) image segmentation is challenging and critical for BUS computer-aided diagnosis (CAD) systems. Many BUS segmentation approaches have been studied in the last two decades, but the performances of most approaches have been assessed using relatively small private datasets with different quantitative metrics, which results in a discrepancy in performance comparison. Therefore, there is a pressing need for building a benchmark to compare existing methods using a public dataset objectively, to determine the performance of the best breast tumor segmentation algorithm available today, and to investigate what segmentation strategies are valuable in clinical practice and theoretical study. In this work, a benchmark for B-mode breast ultrasound image segmentation is presented. In the benchmark, (1) we collected 562 breast ultrasound images and proposed standardized procedures to obtain accurate annotations using four radiologists; (2) we extensively compared the performance of 16 state-of-the-art segmentation methods and demonstrated that most deep learning-based approaches achieved high dice similarity coefficient values (DSC ≥ 0.90) and outperformed conventional approaches; (3) we proposed the losses-based approach to evaluate the sensitivity of semi-automatic segmentation to user interactions; and (4) the successful segmentation strategies and possible future improvements were discussed in details.

## 1. Introduction

Breast cancer occurs with the highest frequency in women among all cancers and is also one of the leading causes of cancer death worldwide [1]. The key to reducing mortality is to find the signs and symptoms of breast cancer at its early stage. In current clinical practice, breast ultrasound (BUS) imaging with computer-aided diagnosis (CAD) system has become one of the most important and effective approaches for breast cancer detection due to its non-invasive, non-radioactive, and cost-effective nature. In addition, it is the most suitable approach for large-scale breast cancer screening and diagnosis in low-resource countries and regions.

CAD systems based on B-mode breast ultrasound (BUS) have been developed to overcome the inter- and intra-variabilities of the radiologists’ diagnoses and have demonstrated the ability to improve the diagnosis performance of breast cancer [2]. Automatic BUS segmentation, extracting tumor region from normal tissue regions of BUS image, is a crucial component in a BUS CAD system. It can change the traditional subjective tumor assessments into operator-independent, reproducible, and accurate tumor region measurements.

Driven by clinical demand, automatic BUS image segmentation has attracted great attention in the last two decades; and many automatic segmentation algorithms have been proposed. The existing approaches can be classified into semi-automatic and fully automatic according to “with or without” user interactions in the segmentation process. In most semi-automatic methods, the user needs to specify a region of interest (ROI) containing the lesion, a seed in the lesion, or an initial boundary. Fully automatic segmentation is usually considered as a top-down framework that models the knowledge of breast ultrasound and oncology as prior constraints and needs no user intervention at all. However, it is quite challenging to develop automatic tumor segmentation approaches for BUS images, due to the poor image quality caused by speckle noise, low contrast, weak boundary, and artifacts. Furthermore, tumor size, shape, and echo strength vary considerably across patients, which prevents the application of strong priors to object features that are important for conventional segmentation methods.

In previous works, most approaches were evaluated by using private datasets and different quantitative metrics (see Table 1), which make the objective and effective comparisons among the methods quite challenging. As a consequence, it remains difficult to determine the best performance of the algorithms available today, what segmentation strategies are accessible to clinic practice and study, and what image features are helpful and useful in improving segmentation accuracy and robustness.

In this paper, we present a BUS image segmentation benchmark including 562 B-Mode BUS images with ground truths, and compare 16 state-of-the-art BUS segmentation methods by using seven popular quantitative metrics. Besides the BUS dataset in this study, three other BUS datasets [24,25,26] were published recently. References [24,25] have many challenging images with small tumors and could be valuable to test algorithm performance in segmenting small tumors; however, [24] has only 163 images, and [25] does not have ground truths for most images. The work of [26] has 763 images, including 133 normal images (without tumors). It is valuable to test algorithms’ robustness in dealing with normal images. However, the three datasets did not use the same standardized process for ground truth generation; therefore, we do not report the performance of the algorithms for them.

We also make the BUS dataset and the performance of the 16 approaches available at http://cvprip.cs.usu.edu/busbench. (1 May 2018) To the authors’ best knowledge, this is the first attempt to benchmark the BUS image segmentation methods. With the help of this benchmark, researchers can compare their methods with other algorithms and find the primary and essential factors for improving the segmentation performance.

The paper is organized as follows: Section 2 gives a brief review of BUS image segmentation approaches; Section 3 illustrates the setup of the benchmark; in Section 4, the experimental results are presented; and the discussions and conclusion are in Section 5 and Section 6, respectively.

## 2. Related Works

Many BUS segmentation approaches have been studied in the last two decades and have proven effective using private datasets. In this section, a brief review of automatic BUS image segmentation approaches is presented. For more details, refer to the survey paper [27]. The BUS image segmentation approaches are classified into five categories: (1) deformable models, (2) graph-based approaches, (3) machine learning-based approaches, (4) classical approaches, and (5) other approaches.

**Deformable models (DMs)**. According to the ways of representing the curves and surfaces, DMs are generally classified into two subcategories: (1) parametric DMs (PDMs) and (2) geometric DMs (GDMs). PDMs-based segmentation approaches focused on generating good initial tumor boundaries. PDMs [3,28,29,30,31,32] were investigated by utilizing different preprocessing methods such as the balloon forces, sticks filter, gradient vector flow (GVF) model, watershed approach, etc. In GDMs-based BUS image segmentation approaches, many methods focused on dealing with the weak boundary and inhomogeneity of BUS images. The authors of [33,34,35,36,37,38] utilized the active contour without edges (ACWE) model, Mumford-Shah technique, signal-to-noise ratio and local intensity value, level set approach, phase congruency, etc. Liu et al. [4] proposed a GDMs-based approach that enforced priors of intensity distribution by calculating the probability density difference between the observed intensity distribution and the estimated Rayleigh distribution. Two major challenges exist in DMs. (1) PDMs-based approaches are sensitive to initial curves and unable to adapt to topological changes of the objects; and (2) GDMs solved the challenge of PDMs, but increased the computational cost greatly.

**Graph-based approaches**. Graph-based approaches gain popularity in BUS image segmentation because of their flexibility and efficient energy optimization. The Markov random field–maximum a posteriori–iterated conditional mode (MRF-MAP-ICM) and the graph cuts or conditional random fields (CRFs) are the two major frameworks in graph-based approaches [27]. Potts model [39] was a common choice for defining the prior energy [40,41]. The authors of [41,42,43] utilized Gaussian distribution to model intensity and texture, and the Gaussian parameters were either from manual selection or from user interactions. 

Graph cuts is a special case of the MRF-MAP modeling, but focuses on binary segmentation. Xian et al. [5] proposed a novel fully automatic BUS image segmentation framework in which the graph cuts energy modeled the information from both the frequency and space domains. Shao et al. [6] modeled a tumor saliency map to exclude non-tumor regions and applied the map to define the data term in graph cuts. Chiang et al. [44] built the graph using image regions, which was initialized by specifying a group of tumor regions and a group of background regions and defined the weight function of the smoothness term (prior energy) using regional intensity difference and edge strength [45]; and the data term was determined online by a pre-trained probabilistic boosting tree (PBT) classifier [46]. In [13], a hierarchical multi-scale superpixel classification framework was proposed to define the data term. The “shrink” problem is a common challenge for all graph-based approaches, which leads to an object boundary that is shorter than the actual one. Normalized cut was proposed to solve this challenge but at the cost of high computation and inflexibility in integrating semantic information [27].

**Machine learning-based approaches**. Both supervised and unsupervised learning approaches have been applied to BUS image segmentation. Unsupervised approaches are simple and fast and commonly utilized as preprocessing to generate candidate image regions. Supervised approaches are good for integrating features at different levels and producing accurate results.

*Clustering*: Xu et al. [47] proposed a BUS image segmentation method by applying the spatial fuzzy c-means (sFCM) [48] to the local texture and intensity features. In [49], FCM was applied to intensities for generating image regions in four clusters. Moon et al. [11] applied FCM to image regions produced by using the mean shift method. Shan et al. [12] extended the FCM and proposed the neutrosophic l-means (NLM) clustering to deal with the weak boundary problem in BUS image segmentation by considering the indeterminacy membership. Clustering approaches are sensitive to initialization and may require a fixed threshold to determine tumor regions. These approaches are usually applied as a preprocessing step to locate rough tumor regions.

*SVM and shallow NNs*: Liu et al. [50] trained a support vector machine (SVM) using local image features to categorize small image lattices (16 × 16) into the tumor or non-tumor classes. Jiang et al. [14] trained Adaboost classifier using 24 Haar-like features [51] to generate a set of candidate tumor regions. Huang et al. [52] proposed an NN-based method to segment 3D BUS images by processing 2D image slices using local image features. Two artificial neural networks (ANNs) to determine the best-possible threshold were trained [53]. Shan et al. [15] trained an ANN to conduct pixel-level classification by using the joint probability of intensity and texture [28] with two new features: the phase in the max-energy orientation (PMO) and radial distance (RD). The ANN had six hidden nodes and one output node. The SVM and shallow NNs for breast tumor segmentation depend on hand-crafted features and may need preprocessing approaches to partition images and post-processing approaches to refine the results.

*Deep Learning*: deep learning-based approaches have been reported to achieve state-of-the-art performance for many medical tasks such as prostate segmentation [54], cell tracking [55], muscle perimysium segmentation [56], brain tissue segmentation [57], breast tumor diagnosis [58], etc. Deep learning models have great potential to achieve good performance due to the ability to characterize large image variations and to learn compact image representations using a sufficiently huge image dataset automatically. Deep learning architectures based on convolutional neural networks (CNNs) were employed in medical image segmentation. U-Net [55], LeNet [59], FCN [60], and SegNet [61] are popular architectures used in BUS image segmentation. Huang et al. [62] combined fuzzy logic with FCN, and the five-layer structure of the breast is utilized to refine the final segmentation results. Huang et al. [19] applied fuzzy logic to five convolutional blocks. It can handle the breast images having no tumors or more than one tumor which was not processed well before. Huang et al. [20] utilized fully convolutional CNNs to identify the tissue layers of the breast and integrated the layer information into a fully connected CRF model to generate the final segmentation results. Shareef et al. [21] proposed the STAN architecture to improve small tumor segmentation. Two encoders were employed in STAN to extract the multi-scale contextual information from different levels of the contracting part. Zhuang et al. [63] proposed the RDAU-Net, which used the dilated residual blocks and attention gates to replace the basic blocks and original skip connections in U-Net, respectively. RDAU-Net improved the overall sensitivity and accuracy of tumor segmentation on BUS images. Guan et al. [64] proposed a semantic context aware network (SCAN), which integrates a location fusion module and context fusion module to detect semantic and contextual features. To segment objects at different scales, Ibtehaz [65] improved U-Net by replacing the convolutional blocks with inception-like blocks and replacing the original skip connections with convolutions operations. Gu et al. [66] proposed the context encoder network (CE-Net) by integrating a context encoder module into a U-Net framework to preserve more spatial information. The DenseU-Net [67] architecture used residual connections and a weighted focal loss function with median frequency balancing to improve the performance of small object detection. Regardless of the unprecedented performance of deep-learning approaches for breast tumor segmentation, two major challenges exist. (1) Most deep learning approaches are ‘blackbox’ and lack essential explainability to justify the results. (2) Popular deep-learning frameworks are vulnerable to adversarial attacks; hence implementing and integrating defense strategies, e.g., RST [68], TREADES [69], and LLR [70], are valuable to improve the adversarial robustness of deep-learning models.

**Classical approaches**: The three most popular classical approaches were applied to BUS image segmentation: thresholding, region growing, and watershed. Thresholding [15,71] was applied to automatically segment breast tumors. Kwak et al. [72] defined the cost of growing a region by modeling common contour smoothness and region similarity (mean intensity and size). Watershed could produce more stable results than thresholding and region-growing approaches. Huang et al. [73] selected the watershed markers based on the grey level and connectivity. Zhang et al. [74] applied watershed to determine the boundaries of the binary image. The markers were set as the connected dark regions. Lo et al. [75] applied watershed and post-refinement based on the grey level and location to generate candidate tumor regions. The classical approaches are simple and fast, but usually were implemented as preprocessing steps to facilitate other approaches.

**Other approaches**: Two interesting approaches are in this category: cell computation [44,45] and cellular automation [22]. In cell computation, the cells are small image regions, and adjacent cells compete with each other to split or merge. Chen et al. [45] defined two types of competitions: Type I and Type II. In Type I competition, two adjacent cells from different regions compete to split one cell from a region and merge it into another region. One cell splits from a multi-cell region and generates a single-cell region in Type II competition. This approach is simple and fast, but it needs user interaction to select the tumor region. In cellular automation (CA), each cell has three components: state, neighbors, and a transition function. A cell’s state updates by using its transition function and the states of its neighboring cells. Liu et al. [22] constructed the transition function by using local texture correlation. It could generate accurate tumor boundaries and did not have the shrink problem in graph cuts. The computation cost for CA to reach a stable state set was quite high.

In Table 1, we list 21 BUS image segmentation approaches published recently.

## 3. Benchmark Setup

### 3.1. BUS Segmentation Approaches and Setup

We obtained permissions from the developers of six BUS segmentation methods [4,5,6,15,21,22] to use their source code. In addition, we implemented 10 deep learning-based approaches: Fuzzy FCN [62], Fuzzy FCN Pyramid [19], FCN [60], U-Net [55], SegNet [61], MultiResUNet [65], CE-Net [66], RDAU Net [63], SCAN [64], and DenseU-Net [67]. Approaches in [22] and [4] are interactive and both need an operator to specify regions of interest (ROIs) manually. All other approaches are fully automatic.

Among the 16 approaches, [4,5,6,15,19,21,22,62] were trained and tested using BUS datasets. [5] is an unsupervised approach and was originally validated using 184 BUS images; and the spatial term weight λ is set to 2.4. Shan et al. [15] utilized a predefined reference point (center of the upper part of the image) for seed generation and pre-trained tumor grey-level distribution for texture feature extraction; we use the same reference point (i.e., image center) and the predefined grey-level distribution provided by the authors; and 10-fold cross-validation is employed to evaluate the overall segmentation performance. Reference [4] is a level set-based segmentation approach and sets the initial tumor boundary by user-specified ROI. The maximum number of iterations is set to 450 as the stopping criterion. Reference [22] is based on cellular automation and uses the pixels on the boundary of the ROI specified by the user as the background seeds and pixels on a cross at the ROI center as the tumor seeds. Reference [6] is a graph-based fully automatic approach, and was originally evaluated using 450 BUS images. In our experiments, we adopt all the parameters from the original papers correspondingly. References [43,48,49,53,54,55,56,57,58] are deep-learning approaches, and 5-fold cross-validation was applied to test the performance. To overcome memory restrictions, we used a batch size of four. They were optimized in the same way as described in their original papers.

### 3.2. Dataset and Ground Truth Generation

Our BUS image dataset has 562 images among women between the ages of 26 to 78 years. The images were originally collected and de-identified by the Second Affiliated Hospital of Harbin Medical University, the Affiliated Hospital of Qingdao University, and the Second Hospital of Hebei Medical University using multiple ultrasound devices: GE VIVID 7 and LOGIQ E9, Hitachi EUB-6500, Philips iU22, and Siemens ACUSON S2000. Since this project only involves historical and de-identified data, the IRB approval was exempt. 

**Figure 1:**
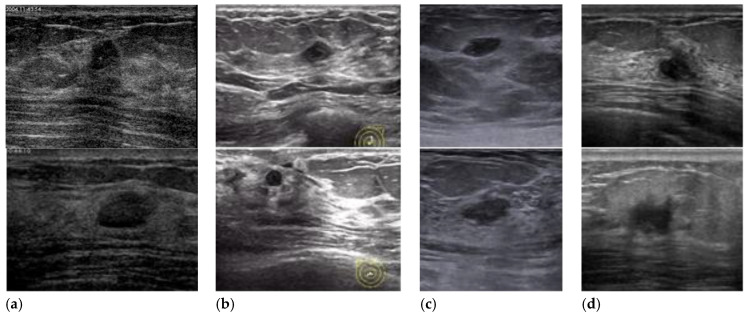
Breast ultrasound images collected using different devices. BUS images produced using (**a**) GE VIVID 7, (**b**) GELOGIQ E9, (**c**) Simens ACUSON S2000, and (**d**) Hitachi EUB-6500.

The images from different sources are valuable for testing the robustness of algorithms. Example images from different devices are shown in Figure 1. Informed consent to the protocol from all patients was acquired. The privacy of the patients is well protected.

**Figure 2:**
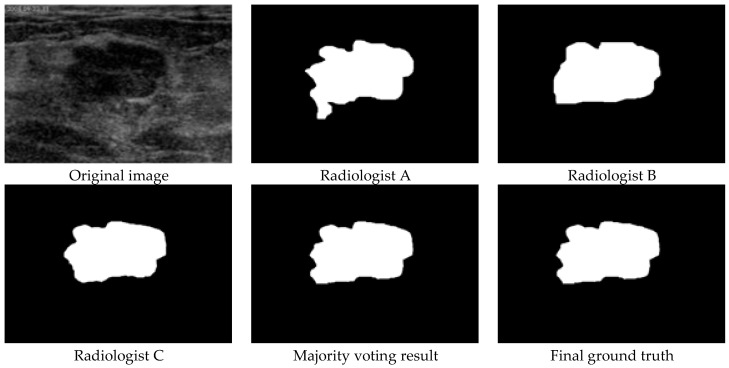
Ground truth generation.

Four experienced radiologists are involved in the ground truth generation; three radiologists read each image and delineate each tumor boundary individually, and the fourth one (senior expert) judges if the majority voting results need adjustments. The ground truth generation has four steps: (1) each of the three experienced radiologists delineate each tumor boundary manually, and three delineation results are produced for each BUS image. (2)Aall pixels inside/on the boundary are viewed as tumor region, outside pixels as background; and majority voting is used to generate the preliminary result for each BUS image. (3) The senior expert reads each BUS image and refers to its corresponding preliminary result to decide if it needs any adjustment. (4) We label the tumor pixel as 1 and the background pixel as 0 and generate a binary and uncompressed image as the ground truth for each BUS image. An example of the ground truth generation is in Figure 2.

### 3.3. Quantitative Metrics

Among the approaches, two of them [4,22] are semi-automatic, and user predefined region of interest (ROI) needs to be set before the segmentation; while the other 14 approaches are fully automatic. The performance of semi-automatic approaches may vary with different user interactions. It is meaningless to compare semi-automatic methods with fully automatic methods; therefore, we will compare the methods in two categories separately. In the evaluation of semi-automatic approaches, we compare the segmentation performances of the two methods using the same set of ROIs and evaluate the sensitivity of the methods to ROIs with different looseness ratio (*LR*) defined by

(1) $$LR=\frac{BD}{BD_{0}}$$

where *BD*_0_ is the size of the bounding box of the ground truth and is used as the baseline, and *BD* is the size of an ROI containing *BD*_0_. We produce 10 groups of ROIs with different *LRs* automatically using the approach described in [76]: move the four sides of an ROI toward the image borders to increase the looseness ratio; and the amount of the move is proportional to the margin between the side and the image border. The *LR* of the first group is 1.1; and the *LR* of each of the other groups is 0.2 larger than that of its previous group.

The method in [15] is fully automatic, it involves neural network training and testing, and a 10-fold cross-validation strategy is utilized to evaluate its performance. Methods in [5,6] need no training and operator interaction. All experiments are performed using a windows-based PC equipped with a dual-core (2.6 GHz) processor and 8 GB memory. The performances of these methods are validated by comparing the results with the ground truths. Both area and boundary metrics are employed to assess the performances of the approaches. The area error metrics include the true positive ratio (TPR), false positive ratio (FPR), Jaccard index (JI), dice similarity coefficient (DSC), and area error ratio (AER)

(2) $$\mathrm{TPR}=\frac{\left|A_{m}\cap A_{r}\right|}{\left|A_{m}\right|}\;$$

(3) $$\mathrm{FPR}=\frac{\left|A_{m}\cup A_{r}-A_{m}\right|}{\left|A_{m}\right|}\;$$

(4) $$\mathrm{JI}=\frac{\left|A_{m}\cap A_{r}\right|}{\left|A_{m}\cup A_{r}\right|}\;$$

(5) $$\mathrm{DSC}=\frac{2\left|A_{m}\cap A_{r}\right|}{\left|A_{m}\right|+\left|A_{r}\right|}$$

(6) $$\mathrm{AER}=\frac{\left|A_{m}\cup A_{r}\right|-\left|A_{m}\cap A_{r}\right|}{\left|A_{m}\right|}\;$$

where *A_m_* is the pixel set in the tumor region of the ground truth, *A_r_* is the pixel set in the tumor region generated by a segmentation method, and |·| indicates the number of elements of a set. TPR, FPR, and AER take values in [0, 1]; and FPR could be greater than 1 and takes value in [0, +∞). Furthermore, Hausdorf error (HE) and mean absolute error (MAE) are used to measure the worst possible disagreement and the average agreement between two boundaries, respectively. Let *C_m_* and *C_r_* be the boundaries of the tumors in the ground truth and the segmentation result, respectively. The HE is defined by

(7) $$\mathrm{HE}\left(C_{m},C_{r}\right)=\max\left\{\underset{x\in C_{m}}{\max}\left\{d\left(x,C_{r}\right)\right\},\underset{y\in C_{r}}{\max\left\{d\left(y,C_{m}\right)\right\}}\right\}\;\;\;$$

where *x* and *y* are the points on the boundaries $C_{m}$ and $C_{r}$, respectively; and *d*(∙,*C*) is the distance between a point and a boundary *C* as

(8) $$d\left(z,C\right)=\underset{k\in C}{\min}\left\{\left||z-k|\right|\right\}$$

where ‖*z − k*‖ is the Euclidean distance between points *z* and *k*; and *d*(*z*,*C*) is the minimum distance between point *z* and all points on *C*. MAE is defined by

(9) $$\mathrm{MAE}\left(C_{m},C_{r}\right)=\frac{1}{2\left(\sum_{x\in C_{m}}\frac{d\left(x,C_{r}\right)}{n_{r}}+\sum_{y\in C_{r}}\frac{d\left(y,C_{m}\right)}{n_{m}}\right)}.\;$$

where $n_{r}$ and $n_{m}$ are the numbers of points on boundaries $C_{r}$ and $C_{m},$ respectively.

The seven metrics above were discussed in [27]. For the first two metrics (TPR and FPR), each of them only measures a certain aspect of the segmentation result, and is not suitable for describing the overall performance; e.g., a high TPR value indicates that most portions of the tumor region are in the segmentation result; however, it cannot claim an accurate segmentation because it does not measure the ratio of correctly segmented non-tumor regions. The other five metrics (JI, DSC, AER, HE, and MAE) are more comprehensive and effective to measure the overall performance of the segmentation approaches and are commonly applied to tune the parameters of the segmentation models [5], e.g., large JI and DSC, and small AER, HE, and MAE values indicate the high overall performance.

Although JI, DSC, AER, HE, and MAE are comprehensive metrics, we still recommend using both TPR and FPR for evaluating BUS image segmentation; since with these two metrics we can discover some hidden characteristics that cannot be found through the comprehensive metrics. Suppose that the algorithm has low overall performance (small JI and DSC, and large AER, HE, and MAE), if FPR and TPR are large, we can conclude that the algorithm has overestimated the tumor region; if both FPR and TPR are small, the algorithm has underestimated the tumor regions. The findings from TPR and FPR can guide the improvement of the algorithms.

## 4. Approach Comparison

In this section, we evaluate 16 state-of-the-art approaches [4,5,6,15,19,21,22,55,60,61,62,63,64,65,66,67]. The 16 approaches are selected based on three criteria: (1) select at least one representative approach for each category except the classic approaches; (2) each approach should achieve good performance in their original dataset; and (3) the source codes of the approaches are available. Fourteen approaches [5,6,15,19,21,55,60,61,62,63,64,65,66,67] are fully automatic, and we compare their average performances by using the seven metrics discussed in Section 3.2; while for semi-automatic approaches [4,22], we also evaluate their sensitivities of the seven metrics with different *LRs*.

### 4.1. Semi-Automatic Segmentation Approaches

**Figure 3:**
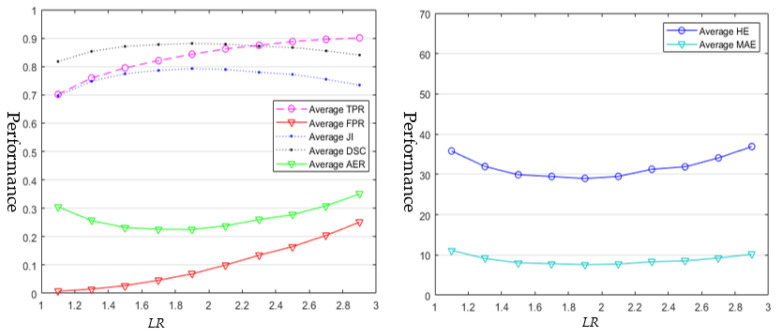
Average segmentation results of [22] using ROIs with diferent looseness ratios (*LRs*).

**Figure 4:**
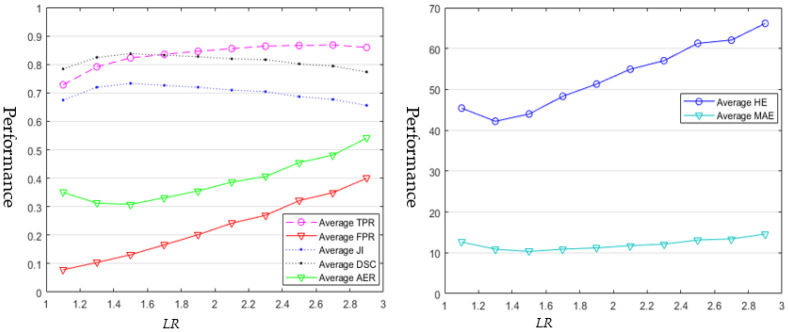
Average segmetation results of [4] using ROIs with different looseness ratios (*LRs*).

Ten ROIs were generated automatically for each BUS image, with an *LRs* range from 1.1 to 2.9 (step size is 0.2). In total, 5620 ROIs were generated for the entire BUS dataset, and we ran each of the semi-automatic segmentation approaches 5620 times to produce the results. All the segmentation results on the ROIs with the same *LR* were utilized to calculate the average TPR, FPR, DSC, AER, HE, and MAE, respectively; and the results of [4] and [22] are shown in Figure 3 and Figure 4, respectively.

The results of [22] are demonstrated in Figure 3. All average JI values are between 0.7 and 0.8; and all average DSC values are between 0.8 and 0.9. The average TPR values are above 0.7 and increase with *LR*s of ROIs; the average JI and DSC values increase firstly, and then decrease; the average FPR values increase with the increasing *LRs*; and the average DSC, HE, and MAE decrease firstly, and then increase. Five metrics (average JI, DSC, AER, HE, and MAE) reach their optimal values at the *LR* of 1.9 (Table 2).

As shown in Figure 4, all the average TPR and DSC values of the method in [4] are above 0.7, and the average JI values vary in the range [0.65, 0.75]. The average TPR values increase with the increasing *LR* values of ROIs. Both the average JI and DSC values tend to increase first and then decrease with the increasing *LR*s of ROIs. FPR, AER, and HE have low average values when the *LR*s are small, which indicates that the high performance of the method in [4] can be achieved by using tight ROIs; however, the values of the three metrics increase almost linearly with the *LR*s of ROIs when the looseness is greater than 1.3; this observation shows that the overall performance of [4] drops rapidly by using large ROIs above a certain level of *LR*. The average MAE values decrease first and then increase and vary with the *LRs* in a small range. Four metrics (average JI, DSC, AER, and MAE) reach their optimal values at the *LR* of 1.5 (Table 2). After 1.5, the increasing ROIs make [4] segment more non-tumor regions into the tumor region (refer to the average FPR curve in Figure 4). The increasing false positive results in the decreasing of the average, JI, and DSC values, and increasing of all other metrics.

As shown in Figure 3 and Figure 4, and in Table 2**,** the two approaches achieve their best performances with different *LRs* (1.5 and 1.9 respectively). We can observe the following facts:

- The works of [4,22] are quite sensitive to the sizes of ROIs.
- The works of [4,22] achieve the best performance when setting them with their optimal *LR*s (1.9 for [22] and 1.5 for [4]).
- The performances of the two approaches drop if the looseness level is greater than a certain value; and the performance of method [22] drops much slower than that of the method in [4].
- Set 1.9 as the optimal *LR* for [22] and 1.5 for [4]; and [22] achieves better average performance than that of [4].
- The running time of the approach in [4] is proportional to the size of the specified ROI, while there is no such relationship of the running time for the approach in [22].
- The running time of the approach in [22] is slower than that of the approach in [4] by one order of magnitude.

### 4.2. Fully Automatic Segmentation Approaches 

The performance of 14 fully automatic approaches is reported in Table 3. Except for methods [5,6,15], the other approaches are deep convolutional neural networks. In general, all deep learning approaches outperform [5,6,15], using the benchmark dataset. The work of [5] achieves better performance than that of the methods in [6,15] on all five comprehensive metrics. The works of [19,22] achieve the lowest average FPR. The method in [15] has the same average TPR value as the method in [5]; however, its average FPR value is much high (1.06), which is almost six times larger than that of the method in [5]; the high average FPR and AER values of the method in [15] indicate that large portions of non-tumor regions are misclassified as tumor regions. The average JIs of all deep learning approaches are above 0.8 except FCN-AlexNet; and [62] achieved the best average JI performance. Table 3 also shows the average optimal performances of [4] and [22] at the *LR*s of 1.5 and 1.9, respectively.

## 5. Discussions

Many semi-automatic segmentation approaches are utilized for BUS image segmentation [27]. User interactions (setting seeds and/or ROIs) are required by these approaches and could be useful for segmenting BUS images with extremely low quality. As shown in Table 3, the two interactive approaches could achieve very good performance if the ROI is set properly.

Figure 3 and Figure 4 also demonstrate that the two semi-automatic approaches achieve varying performances using different sizes of ROIs. Therefore, the major issue in semi-automatic approaches is to determine the best ROIs/seeds. HOwever, such issue has been neglected before completely. Most semi-automatic approaches focused only on improving segmentation performance by designing complex features and segmentation models, but failed to consider user interaction as an important factor that could affect the segmentation performance. Hence, we recommend that researchers should consider such issues when they develop semi-automatic approaches. Two possible solutions could be employed to solve this issue. First, for a given approach, we could choose the best LR by running experiments on a given BUS image training set (like Section 4.1) and apply the LR to the test set. Second, like the interactive segmentation approach in [76], we could bypass this issue by designing segmentation models less sensitive to user interactions. 

Fully automatic segmentation approaches have many good properties, such as operator-independence and reproducibility. The key strategy shared by many successful fully automatic approaches is to localize the tumor ROI accurately by modeling domain knowledge. The authors of [15] localized tumor ROI by formalizing the empirical tumor location, appearance, and size; [24] generated tumor ROI by finding adaptive reference position; and in [6], the ROI was generated to detect the mammary layer of BUS image, and the segmentation algorithm only detected the tumor in this layer. However, in many fully automatic approaches, the performance heavily depends on hand-crafted features and some inflexible constraints, e.g., [15] utilized a fixed reference position to rank the candidate regions in the ROI localization process. Table 3 demonstrates that deep learning approaches outperform all traditional approaches. It is worth noting that deep learning approaches have limitations in segmenting small breast tumors [21].

As shown in Table 3, using the benchmark dataset, the approaches of [4,5,6,15,22] cannot achieve the performances reported in the original papers. The average JI of [5] is 14% less than the original average JI; the average FPR of [15] is 87% higher than the original value; the average TPR of [6] is 17% less than its reported value; and the average JI values of [22] and [4] are 17% and 10% lower than the reported values, respectively. There are two possible reasons: (1) many previous approaches were trained and tested using small BUS datasets from a single source or ultrasound machine, and the images lacked variations. However, the proposed benchmark dataset was collected from multiple ultrasound machines with images of large variations; and (2) existing approaches lack the robustness to adapt to the differences in data distributions.

As shown in Table 1, many quantitative metrics exist for evaluating the performances of BUS image segmentation approaches. In this paper, we applied seven metrics [27] to evaluate BUS image segmentation approaches. As shown in Figure 3 and Figure 4, average JI, DSC, and AER have the same trend, and each of them is sufficient to evaluate the area error comprehensively. To further improve the performance of current approaches, multitask learning and classifier fusion strategies [77] could be future directions.

## 6. Conclusions

In this paper, we established a BUS image segmentation benchmark and presented and discussed the results of 16 state-of-the-art segmentation approaches; two of them are semi-automatic, and others are fully automatic. The BUS dataset contains 562 BUS images collected and has significant variations in image contrast, brightness, and noise level. The quantitative analysis of the considered approaches highlights the following findings.

- As shown in Table 3, by using the benchmark, no approaches in this study can achieve the same performances reported in their original papers, which demonstrates the models’ poor capability/robustness to adapt to BUS images from different sources.
- The two semi-automatic approaches are quite sensitive to user interaction (See Figure 3 and Figure 4). We recommend researchers evaluate the sensitivity of their semi-automatic approaches to user interactions in the future.
- Deep learning approaches outperform all conventional approaches using our benchmark dataset; but the explainability and robustness of existing approaches still need to be improved.
- The quantitative metrics such as JI, DSC, AER, HE, and MAE are more comprehensive and effective to measure the overall segmentation performance than TPR and FPR; however, TPR and FPR are also useful for developing and improving algorithms.

In addition, the benchmark should be and will be expanded continuously.

## Figures and Tables

**Table 1:** Recently published approaches.

Article	Type	Year	Category	Dataset Size/Availability	Metrics
Kuo, et al. [3]	S	2014	Deformable models	98/private	DSC
Liu, et al. [4]	S	2010	Level set-based	79/private	TP, FP, SI
Xian, et al. [5]	F	2015	Graph-based	184/private	TPR, FPR, SI, HD, MD
Shao, et al. [6]	F	2015	Graph-based	450/private	TPR, FPR, SI
Huang, et al. [7]	S	2014	Graph-based	20/private	ARE, TPVF, FPVF, FNVF
Xian, et al. [8]	F	2014	Graph-based	131/private	SI, FPR, AHE
Gao, et al. [9]	S	2012	Normalized cut	100/private	TP, FP, SI, HD, MD
Hao, et al. [10]	F	2012	CRF + DPM	480/private	JI
Moon, et al. [11]	S	2014	Fuzzy C-means	148/private	Sensitivity and FP
Shan, et al. [12]	F	2012	Neutrosophic L-mean	122/private	TPR, FPR, FNR, SI, HD, and MD
Hao, et al. [13]	F	2012	Hierarchical SVM + CRF	261/private	JI
Jiang, et al. [14]	S	2012	Adaboost + SVM	112/private	Mean overlap ratio
Shan, et al. [15]	F	2012	Feedforward neural network	60/private	TPR, FPR, FNR, HD, MD
Pons, et al. [16]	S	2014	SVM + DPM	163/private	Sensitivity, ROC area
Yang, et al. [17]	S	2012	Naive Bayes classifier	33/private	FP
Torbati, et al. [18]	S	2014	Feedforward Neural network	30/private	JI
Huang, et al. [19]	F	2020	Deep CNNs	325/private + 562/public	TPR, FPR, JI, DSC, AER, AHE, AME
Huang, et al. [20]	F	2018	Deep CNNs + CRF	325/private	TPR, FPR, IoU
Shareef, et al. [21]	F	2020	Deep CNNs	725/public	TPR, FPR, JI, DSC, AER, AHE, AME
Liu, et al. [22]	S	2012	Cellular automata	205/private	TPR, FPR, FNR, SI
Gómez, et al. [23]	S	2010	Watershed	50/private	Overlap ratio, NRV and PD

F: fully automatic, S: semi-automatic, SVM: support vector machine, CRF: conditional random field, DPM: deformable part model, CNNs: convolutional neural networks, TP: true positive, FP: false positive, SI: similarity index, HD: Hausdorff distance, MD: mean distance, DSC: Dice similarity, JI: Jaccard index, ROC: Receiver operating characteristic, ARE: average radial error, TPVF: true positive volume fraction, FPVF: false positive volume fraction, FNVF: false negative volume fraction, NRV: normalized residual value, PD: proportional distance, TPR: true positive ratio, FPR: false positive ratio, FNR: false negative ration, and IoU: Intersection over union.

**Table 2:** Quantitative results of [4,22] using 10 *LR*s of ROI.

	Metrics	*LRs*	Area Error Metrics					Boundary Error Metrics		Time
Methods			Ave. TPR	Ave. FPR	Ave. JI	Ave. DSC	Ave. AER	Ave. HE	Ave. MAE	Ave. Time (s)
[4]		1.1	0.73 (0.23)	**0.08** (0.09)	0.67 (0.20)	0.78 (0.18)	0.35 (0.22)	45.4 (31.6)	12.6 (10.9)	**18**
		1.3	0.79 (0.18)	0.10 (0.12)	0.72 (0.16)	0.82 (0.14)	0.31 (0.19)	**42.2 (28.0)**	10.9 (8.9)	22
		1.5	0.82 (0.15)	0.13 (0.14)	**0.73** (0.14)	**0.84** (0.11)	**0.31 (0.18)**	44.0 (28.3)	**10.4 (7.5)**	27
		1.7	0.83 (0.15)	0.17 (0.18)	**0.73** (0.14)	0.83 (0.12)	0.33 (0.20)	48.3 (32.2)	10.9 (8.0)	27
		1.9	0.85 (0.14)	0.20 (0.21)	0.72 (0.14)	0.83 (0.12)	0.36 (0.23)	51.3 (35.3)	11.2 (7.9)	30
		2.1	0.86 (0.14)	0.24 (0.25)	0.71 (0.15)	0.82 (0.13)	0.39 (0.27)	54.9 (38.8)	11.7 (8.4)	30
		2.3	0.86 (0.13)	0.27 (0.28)	0.70 (0.15)	0.82 (0.12)	0.41 (0.29)	57.0 (41.7)	12.1 (8.8)	36
		2.5	**0.87** (0.14)	0.32 (0.33)	0.69 (0.16)	0.80 (0.13)	0.46 (0.34)	61.3 (44.2)	13.1 (10.5)	39
		2.7	**0.87** (0.14)	0.35 (0.36)	0.68 (0.17)	0.79 (0.14)	0.48 (0.36)	62.1 (43.3)	13.4 (9.5)	40
		2.9	0.86 (0.17)	0.40 (0.41)	0.66 (0.19)	0.77 (0.17)	0.54 (0.44)	66.2 (46.1)	14.6 (10.7)	44
[22]		1.1	0.70 (0.10)	**0.01** (0.02)	0.70 (0.09)	0.82 (0.07)	0.31 (0.09)	35.8 (17.0)	11.1 (5.3)	487
		1.3	0.76 (0.09)	0.02 (0.03)	0.75 (0.08)	0.85 (0.06)	0.26 (0.09)	32.0 (15.6)	9.1 (4.6)	467
		1.5	0.79 (0.08)	0.03 (0.04)	0.77 (0.08)	0.87 (0.05)	**0.23** (0.09)	29.9 (15.0)	8.1 (4.2)	351
		1.7	0.82 (0.09)	0.05 (0.06)	**0.79** (0.09)	**0.88** (0.06)	**0.23** (0.10)	29.5 (16.5)	7.8 (4.8)	341
		**1.9**	0.84 (0.09)	0.07 (0.07)	**0.79** (0.09)	**0.88** (0.06)	**0.23** (0.11)	**29.0** (17.0)	7.6 (5.3)	336
		2.1	0.86 (0.08)	0.10 (0.09)	**0.79** (0.10)	**0.88** (0.07)	0.24 (0.13)	29.5 (18.4)	7.7 (5.2)	371
		2.3	0.87 (0.09)	0.13 (0.12)	0.78 (0.11)	0.87 (0.08)	0.26 (0.16)	31.3 (21.9)	8.3 (6.4)	343
		2.5	0.89 (0.09)	0.16 (0.14)	0.77 (0.11)	0.87 (0.08)	0.28 (0.17)	31.9 (20.1)	8.5 (6.1)	365
		2.7	**0.90** (0.09)	0.20 (0.15)	0.75 (0.11)	0.85 (0.08)	0.31 (0.18)	34.1 (20.2)	9.2 (5.9)	343
		2.9	**0.90** (0.10)	0.25 (0.18)	0.73 (0.12)	0.84 (0.10)	0.35 (0.22)	36.9 (21.8)	10.2 (6.7)	388

The values in ‘( )’are the standard deviations; and the best performance in each column is highlighted in bold.

**Table 3:** Overal performance of all approaches.

	Metrics	Area Error Metrics					Boundary Error Metrics		Time
Methods		Ave. TPR	Ave. FPR	Ave. JI	Ave. DSC	Ave. AER	Ave. HE	Ave. MAE	Ave. Time (s)
FCN-AlexNet [60]		**0.95**/--	0.34/--	0.74/--	0.84/--	0.39/--	25.1/--	7.1/--	5.8
SegNet [61]		0.94/--	0.16/--	0.82/--	0.89/--	0.22/--	21.7/--	4.5/--	12.1
U-Net [55]		0.92/--	0.14/--	0.83/--	0.90/--	0.22/--	26.8/--	4.9/--	2.15
CE-Net [66]		0.91/--	0.13/--	0.83/--	0.90/--	0.22/--	21.6/--	4.5/--	2.0
MultiResUNet [65]		0.93/--	0.11/--	0.84/--	0.91/--	0.19/--	**18.8**/--	4.1/--	6.5
RDAU NET [63]		0.91/--	0.11/--	0.84/--	0.91/--	0.19/--	19.3/--	4.1/--	3.5
SCAN [64]		0.91/--	0.11/--	0.83/--	0.90/--	0.20/--	26.9/--	4.9/--	4.1
DenseU-Net [67]		0.91/--	0.16/--	0.81/--	0.88/--	0.25/--	25.3/--	5.5/--	3.5
STAN [21]		0.92/--	0.09/--	0.85/--	0.91/--	0.18/--	18.9/--	**3.9**/--	5.8
Xian, et al. [5]		0.81/0.91	0.16/0.10	0.72/0.84	0.83/--	0.36/--	49.2/24.4	12.7/5.8	3.5
Shan, et al. [15]		0.81/0.93	1.06/0.13	0.60/--	0.70/--	1.25/--	107.6/18.9	26.6/5.0	3.0
Shao, et al. [6]		0.67/0.81	0.18/0.12	0.61/0.74	0.71/--	0.51/--	69.2/50.2	21.3/13.4	3.5
Fuzzy FCN [62]		0.94/--	0.08/--	**0.88**/--	0.92/--	**0.14**/--	19.8/--	4.2/--	6.0
Huang, et al. [19]		0.93/0.93	**0.07**/0.07	0.87/0.87	**0.93**/0.93	0.15/0.15	26.0/26.0	4.9/4.9	6.5
Liu, et al. [4] *LR* = 1.5		0.82/0.94	0.13/0.08	0.73/0.87	0.84/--	0.31/--	44.0/26.3	10.4/--	27.0
Liu, et al. [22] *LR* = 1.9		0.84/0.94	**0.07**/0.07	0.79/0.88	0.88/--	0.23/--	29.0/25.1	7.6/--	336.0

The values before the slashes are approaches’ performances on the proposed dataset, and after the slashes are their performances reported in the original publications. Notation ‘--’ indicates that the corresponding metric was not reported in the original paper. The best performance in each column is highlighted in bold.

## Data Availability

The benchmark dataset is available at http://cvprip.cs.usu.edu/busbench (1 May 2018).
